# A life course approach to physical capability: findings from the HALCyon research programme

**DOI:** 10.1186/1753-6561-7-S4-S4

**Published:** 2013-08-23

**Authors:** Diana Kuh

**Affiliations:** 1 University College London (UCL), Medical Research Council (MRC) Unit for Lifelong Health and Ageing, London, UK

## 

Healthy Ageing across the Life Course (HALCyon) (http://www.halcyon.ac.uk), funded by the UK New Dynamics of Ageing cross council research programme, brought together investigators on nine cohort studies covering 30,000 participants born between 1921 and 1958, to investigate how healthy ageing is affected by factors operating across the whole of life. Our focus is on three domains of healthy ageing: (1) physical and cognitive capability (2) psychological and social wellbeing, and (3) biological ageing at the cellular and physiological system levels.

This presentation focused on the lifetime determinants and consequences of physical capability, and the capacity to undertake the physical tasks of daily living. First we showed that objective measures of physical capability, such as grip strength, timed standing balance and chair rises, and walking speed were reliable indicators of ageing by carrying out two systematic reviews that revealed that reduced performance was consistently associated with subsequent mortality and morbidity, as well as reduced wellbeing. With harmonised HALCyon datasets we showed that capability declines with age, and that gender differences in grip strength diminish with increasing age. One question commonly asked is whether ‘it all goes together when it goes’. While strong cross sectional studies show that physical and cognitive capability are strongly correlated, we identified only seven that had investigated change in fluid cognition with change in physical capability; findings were not sufficiently strong or consistent to support a common cause mechanism.

HALCyon research has shown that early life factors, such as birth weight, physical and cognitive development and childhood socioeconomic circumstances are associated with later life capability, either through maximising peak level of function at maturity or its rate of decline. In addition greater adult adiposity was associated with worse capability; the detrimental impact was greatest in the highest two fifths of BMI, and stronger in women than men. Finally there was little evidence that capability was affected by genetic factors or change in telomere length, but there were associations with cortisol levels, measured prospectively and cross-sectionally, which suggest that the ability to mount a good stress-induced response may be a marker of a more reactive and healthier HPA axis with implications for functional ageing.

The main implication of these findings is that health surveillance earlier in life may be able to identify those most at risk of accelerated ageing so that interventions to maintain physical capability and prevent future disability and frailty can be instigated.

